# Transcarotid artery revascularization

**DOI:** 10.1093/bjs/znac421

**Published:** 2022-12-01

**Authors:** Gert J de Borst

**Affiliations:** Department of Vascular Surgery G04.129, University Medical Center Utrecht, Utrecht, The Netherlands

Transcarotid artery revascularization (TCAR) is a hybrid procedure that involves stenting of the common and internal carotid arteries via remote surgical access to the common carotid artery. The United States Food and Drug Administration (FDA) approved TCAR in 2015 as an additional endovascular procedural option to treat patients with carotid artery stenosis. Approval was authorized without supporting evidence from a randomized clinical trial (RCT), under the stipulation that all procedures be entered into the Vascular Quality Initiative (VQI) registry, which captures more than 95 per cent of TCARs performed in the US. Despite a lack of level 1 evidence, TCAR has been rapidly adopted in the US, with approximately 21 000 implants across nearly 500 centres. In 2022 the FDA expanded the indications for a TCAR system (Enroute; Silk Road Medical, Sunnyvale, California, USA) to include patients at standard risk. Previously, TCAR was approved for use only in patients with anatomical or physiological criteria that put them at higher risk of procedural complications with other carotid revascularization options (standard carotid stenting (CAS) or carotid endarterectomy (CEA)).

In Europe, the availability of TCAR was limited to a small number of centres and, recently, Silk Road Medical decided to completely withdraw TCAR from the European market.

To understand the rationale for TCAR it is necessary to review the underlying scientific evidence. Transfemoral carotid artery stenting (TFCAS) is associated with higher 30-day stroke rates than CEA in both symptomatic and asymptomatic patients^1^. TFCAS is also associated with a four times higher procedural risk of new ischaemic brain lesions measured by diffusion-weighted MRI (DW-MRI)^1,2^. Of importance, without exception, all large randomized trials have consistently shown that, beyond the 30-day period, the long-term risk of any stroke following either TFCAS or CEA appears to be virtually the same^1^. The key message, therefore, is that the magnitude of the initial procedural risk remains the key factor in determining the optimal revascularization technique^3^.

The higher procedural stroke risk after CAS is believed to be secondary to intraprocedural embolization, because of surface thrombus and aortic arch manipulation. TCAR was initially developed to reduce the number of embolic complications associated with transfemoral access. TCAR was designed to avoid aortic arch manipulation, by using direct carotid exposure paired with cerebral blood flow reversal to minimize the embolic potential^4^.

Several cohort series and two single-arm trials have claimed favourable procedural stroke and death rates for TCAR, as compared to TFCAS. The technical success rates were indisputably high (98 per cent) and the 30-day mortality and stroke rates remarkably low^5,6^. Prospective research is limited to single-arm investigations in the ROADSTER-1 and ROADSTER-2 studies. ROADSTER-1 was the first multicentre investigation of the ENROUTE transcarotid neuroprotection system (NPS), using a variety of stents. ROADSTER-2 was then performed to evaluate the efficacy of the NPS with a single (ENROUTE) transcarotid stent. In 155 patients deemed to be high risk (77 per cent asymptomatic), none suffered a perioperative or 1-year ipsilateral stroke. The authors, therefore, claimed the safety of TCAR in high-risk patients. However, owing to the heterogenous population and the fact that only one in five eligible patients actually participated in follow-up beyond the in-hospital phase, the conclusions appear shaky^6^.

Findings from the ROADSTER cohorts have been corroborated by the TCAR surveillance project (TSP). The TSP retrospectively examined TCAR stratified by preprocedural symptom status. In 18 477 patients, there was a 2.8 odds ratio of in-hospital stroke/death in patients with a recent stroke, as compared to asymptomatic patients. While there is a clear need for outcome data in selective symptomatic patients only, VQI data are limited to in-hospital endpoints, which underestimate procedural risks by 20–25 per cent, as compared to 30-day results, making direct comparison with 30-day outcomes after TFCAS or CEA less robust^7^.

Significant evidence gaps for the role of TCAR remain^4,8^. No RCT has ever compared TCAR with CEA or TFCAS directly; registry cohorts evaluating TCAR have been dominated by asymptomatic patients; there has been no direct comparison of TCAR *versus* CEA in selectively symptomatic patients (those who have the highest benefit from safe revascularization); most clinical data have been derived from company-sponsored single-arm studies; up to one-third of patients are anatomically unsuitable for TCAR due to calcification or tortuosity and exclusion criteria are under-reported; almost three-quarters of all available data (3/18 studies) have originated from the VQI registry; the quality of the studies reporting TCAR has generally been poor, with one-third classified as moderate and the remaining two-thirds (*n* = 12) as poor quality; no less than seven reviews and meta-analyses have been published, most of which have omitted a discussion of the uncertainties around the effectiveness of the TCAR procedure.

Surface thrombus and embolization are responsible for the significantly higher rates of new MR-DWI lesions seen after CAS. DWI lesions have been associated with increased risk of future cerebrovascular events^2,9^. A small pilot study (TCAR *versus* CEA *versus* TFCAS; 16 *versus* 10 *versus* 8 patients) of embolization rates with intraoperative transcranial Doppler found that TCAR was associated with a significantly lower rate of embolization compared with TFCAS but not with CEA. No study has ever compared procedural embolization of TCAR and CEA using DW-MRI.

Based on the above limitations, clinical practice guidelines therefore still recommend CEA as the first-choice intervention, especially in patients who have recently had symptomatic carotid disease. To corroborate the encouraging VQI data with other real-life prospective audits, data submission has been encouraged^10^.

One of the limitations of TCAR is that it is not widely available outside of the US and Asian markets, which may be the result of logistical and economic factors, with CEA being significantly cheaper.

While carotid stenting has evolved over the past two decades, CEA for now remains the safer option of the two for the majority of patients, and across all periods after the onset of symptoms. However, while we mostly debate a ‘one treatment option only’ concept, it is clear that surgery and stenting have complementary roles. It is essential that these roles are determined by high-quality evidence. The vascular community should learn lessons from the introduction of other technologies^11,12^. At this stage, TCAR requires transparent reporting of outcomes (whether positive or negative) and details about how and why the intervention is modified as experience is gained^13^. The US-based CREST investigators recently proposed a CREST-3 multicentre 30-day endpoint trial comparing TCAR to CEA or TFCAS (G. Howard, personal communication). The proposal was abandoned by industry sponsors and we may only hope for alternative, high-quality prospective studies.

It is obvious that independent RCTs comparing TCAR with CEA in recently symptomatic patients are required to establish the true place of TCAR in carotid revascularization. The key challenge will be to demonstrate that TCAR can be performed safely in the first 14 days after the onset of neurological symptoms, with procedural risks comparable with CEA. Only then can the vascular community and patients embrace TCAR as a genuine alternative treatment option.
